# Case report: Isoflurane therapy in a case of status asthmaticus requiring extracorporeal membrane oxygenation

**DOI:** 10.3389/fmed.2022.1051468

**Published:** 2022-11-08

**Authors:** Brendan Gill, Jason L. Bartock, Emily Damuth, Nitin Puri, Adam Green

**Affiliations:** Department of Critical Care Medicine, Cooper University Health Care, Camden, NJ, United States

**Keywords:** ECMO–extracorporeal membrane oxygenation, status asthmaticus, isoflurane, venovenous ECMO, asthma, respiratory acidosis, bronchospasm, anesthetics: volatile: halothane sevoflurane

## Abstract

Volatile anesthetics have been described as a rescue therapy for patients with refractory status asthmaticus (SA), and the use of isoflurane for this indication has been reported since the 1980s. Much of the literature reports good outcomes when inhaled isoflurane is used as a rescue therapy for patients for refractory SA. Venovenous (VV) extracorporeal membrane oxygenation (ECMO) is a mode of mechanical circulatory support that is usually employed as a potentially lifesaving intervention in patients who have high risk of mortality, primarily for underlying pulmonary pathology. VV ECMO is usually only considered in cases where patients gas exchange cannot be satisfactorily maintained by conventional therapy and mechanical ventilation strategies. We report the novel use of isoflurane delivered systemically as treatment for severe refractory SA in a patient on VV ECMO. A 51-year-old male with a history of asthma was transferred from another institution for management of severe SA. He was intubated at the referring hospital after failing non-invasive ventilation. Initial arterial blood gas (ABG) showed pH 7.21, partial pressure of carbon dioxide (PCO_2_) >95 mmHg, and partial pressure of oxygen (PaO_2_) 60 mmHg. VV ECMO was initiated on hospital day (HD) 1 due to refractory respiratory acidosis. After ECMO initiation, acid-base status improved, however, severe bronchospasm persisted and intrinsic positive end expiratory pressure (PEEP) was measured at 18 cm H_2_O. Systemic paralysis was employed, respiratory rate (RR) was reduced to 4 breaths per minute. This degree of bronchospasm did not allow for ECMO weaning. On HD 5, the patient received systemic isoflurane *via* the ECMO circuit for 20 h. The following morning, intrinsic PEEP was 4 cm H_2_O, and wheezing improved. He was decannulated from VV ECMO on HD 10 and extubated on HD 17. Inhaled isoflurane therapy in patients on VV ECMO for refractory SA has shown good results, but requires delivery of the medication *via* anesthesia ventilators. Our case highlights an effective alternative, systemic delivery of anesthetic *via* the ECMO circuit, as it is often difficult and dangerous to transport these patients to the operating room (OR) or have an intensive care unit (ICU) room adjusted to accommodate an anesthesia ventilator.

## Introduction

### Status asthmaticus overview

Status asthmaticus (SA) is defined as the condition of a patient in progressive respiratory failure due to asthma, in whom conventional forms of therapy have failed (1). Approximately 10% of the world's population suffers from asthma, and there is a 15% disease burden in the United States over the last two decades (2). It is estimated that 3–16% of hospitalized adult patients with asthma progress to respiratory failure requiring ventilator support. Mortality is reported at approximately 10% in ICU patients admitted with SA (3), and reported to be as high as 21% in intubated patients (4).

### Therapeutic approach to status asthmaticus

The standard approach to treating severe asthma exacerbations include the use of short-acting inhaled beta-agonists with a preference for albuterol due to higher beta-2 selectivity (5) along with systemic corticosteroids such as methylprednisolone (6). Other adjunctive pharmacotherapy include inhaled anticholinergics (ipratropium) and intravenous magnesium (7).

### Ventilator management in status asthmaticus

A small percentage (2–4%) of those with SA will require mechanical ventilation due to encephalopathy, inability to oxygenate or ventilate, or muscle fatigue (8, 9). The decision to intubate should be made clinically, and should be considered in patients with increasing lethargy, use of accessory muscles, change in posture or speech, and/or decreasing rate and depth of respiration (10). After initiation of mechanical ventilation, it is important to monitor airway resistance and pulmonary hyperinflation, titrate FiO_2_ to maintain a SpO_2_ 90–92% and adjust minute ventilation to maintain a pH >7.25. To avoid intrinsic PEEP, a strategy of permissive hypercapnia is employed by reducing tidal volumes (5–7 mL/kg), lowering respiratory rate (10–12 breaths per min) and extending expiratory time through adjustment of I:E ratio (I:E 1:3–1:4) (11).

Volume-controlled modes are recommended due to the delivery of constant flow, which can help decrease peak airway pressures. Pressure-controlled modes allow for control of peak airway pressures, however, in asthma the high peak airway pressure is due to airway resistance rather than reduced compliance. In the absence of intrinsic PEEP, plateau pressure should only be mildly elevated, as it provides the best estimate of alveolar distending pressure when esophageal pressure monitoring is unavailable. Elevation of plateau pressure >25 cm H20 should raise concern for severe dynamic hyperinflation, which can cause life-threatening hemodynamic instability and barotrauma. Minimal PEEP should be utilized to prevent intrinsic PEEP and not contribute to hyperinflation.

Patients who are intubated for status asthmaticus should be deeply sedated, and paralyzed if necessary, to prevent any patient-ventilator dyssynchrony.

### Advanced therapies for status asthmaticus

VV ECMO is a form of mechanical circulatory support reserved for those with respiratory failure refractory to conventional treatment (12). Large cannulas are peripherally placed in central veins allowing for the removal and return of venous blood as it is passed through a membrane serving to oxygenate and remove carbon dioxide through centrifugal forces. The use of VV ECMO in status asthmaticus is rare and only reserved for those who have inadequate gas exchange with mechanical ventilation or unsafe airway pressures. Unlike in acute respiratory distress syndrome (ARDS), the criteria for cannulation are less concrete. This is mostly due to the significantly fewer cases. However, those who require ECMO support tend to have favorable outcomes with a survival reported at 84% (13). While selection criteria vary from center to center, common exclusion criteria include age, presence of chronic comorbidities or concern for neurological injury. Potential complications include major bleeding, thromboses, infection, renal failure, pulmonary and CNS hemorrhage among many others. The use of ECMO in asthma patients allows the lungs to rest, thus providing time for bronchiolar relaxation until the bronchospasm has subsided. While imposing risk, ECMO has been used successfully as an early adjunct to therapy in patients whose gas exchange cannot otherwise be satisfactorily maintained by conventional therapy and ventilation strategies (14).

Inhaled volatile anesthetics have been cited as a rescue therapy for severe refractory status asthmaticus (15–18). There are multiple postulated mechanisms regarding how these agents work, including direct relaxation of bronchial smooth muscle, inhibition of the release of inflammatory mediators, beta adrenergic receptor stimulation, reduction of vagal tone and vagal-mediated reflexes and antagonism of the effects of histamine and methacholine (19–21). The use of inhaled anesthetics in patients requiring ECMO has been reported more recently, both in severe refractory SA and acute respiratory distress syndrome (ARDS) (19, 22); however, the literature is limited. In previously reported cases of utilization of inhaled volatile anesthetics for severe SA, the anesthetics were delivered *via* the inhalational route utilizing an anesthesia ventilator (19, 23). In the case presented here, isoflurane was administered systemically *via* the ECMO circuit. To our knowledge, this is the first case in the literature to describe this route of isoflurane delivery.

## Case

A 51-year-old male with no baseline functional limitations and a medical history of asthma and tobacco use disorder initially presented to an outside hospital (OSH) with an acute asthma exacerbation. His outpatient asthma regimen consisted of tiotropium, montelukast, an albuterol inhaler as needed, and prednisone 10 mg daily. He was started on non-invasive positive pressure ventilation at the referring hospital, but required endotracheal intubation for worsening respiratory distress. Post intubation, there was significant difficulty with ventilation due to severe bronchospasm. Despite neuromuscular blockade and attempts at using a variety of mechanical ventilation modes, there was little improvement in his respiratory acidosis. Pharmacological treatment included systemic steroids, bronchodilators, ketamine, intramuscular epinephrine, and racemic epinephrine. The patient was transferred for escalation of care and consideration for VV ECMO.

On arrival to our institution, the patient was noted to have diffuse wheezing. He required continued deep sedation and paralysis for ventilator dyssynchrony. His initial arterial blood gas revealed a pH of 7.21, PCO_2_ > 95 mmHg and a PaO_2_ 60 mmHg on an FiO2 of 40% on pressure control ventilation with an inspiratory pressure of 30 cmH_2_O and a PEEP of 5 cm H_2_O. Due to high intrinsic PEEP (18 cm H_2_O), he was changed to pressure regulated volume control with a tidal volume of 500 mL, respiratory rate of 12 and PEEP of 5 improving his intrinsic PEEP to 12 cm H_2_O.

His respiratory acidosis worsened requiring initiation of VV ECMO on hospital day 1.

VV ECMO criteria used for cannulation included refractory respiratory acidosis with a PCO2 > 60 mmHg despite optimal ventilator management. He was cannulated with a 25 french drainage cannula in the right femoral vein, and a 20 french return cannula in the right internal jugular vein. In order to avoid over correction of his PCO_2_, as this has been linked to poor neurological outcomes (24), the initial sweep gas flow was set at 2 L/min with ventilator settings of PRVC set to a TV 350 mL (4.7 cc/kg IBW), RR 8 breaths per minute and a PEEP of 5 cm H_2_O. Post cannulation ABG demonstrated a pH of 7.32, PcO_2_ 86 mmHg and a PaO_2_ 135 mmHg.

The patient continued to have evidence of severe bronchospasm with wheezing and poor air movement throughout all lung fields and ongoing intrinsic PEEP despite excessive expiratory time. A brief trial of Heliox was utilized but was stopped after no clinical improvement. He was continued on short-acting bronchodilators, systemic and inhaled corticosteroids, montelukast along with chest physiotherapy to avoid atelectasis. The sedation approach included a combination of propofol, ketamine, midazolam, and fentanyl along with periods of neuromuscular blockade. There was improvement in his intrinsic PEEP, but he continued to not tolerate increases in native minute ventilation *via* mechanical ventilation in an attempt to wean sweep gas flow. Given overall lack of significant clinical improvement, a multi-disciplinary team including anesthesia, critical care, perfusion, and pulmonary medicine met to discuss the role of inhaled anesthetics (isoflurane). The patient was initiated on isoflurane *via* the ECMO circuit on hospital day 5.

An isoflurane vaporizer was attached to the ECMO circuit and a line was run from the vaporizer to the ECMO oxygenator. A separate waste anesthesia gas line was run from the circuit to the waste chamber. A separate port at the bottom of the membrane oxygenator served as an open vent to draw air into the oxygenator and not allow air or waste anesthesia gas to escape into the atmosphere, risking staff exposure. Vaporized isoflurane was instilled into the ECMO circuit at an initial concentration of 0.8%, and incrementally increased to 1.2%, and did not go above this (due to safety concerns for patient and providers). This medication was continued for 20 h.

**Figure 1:**
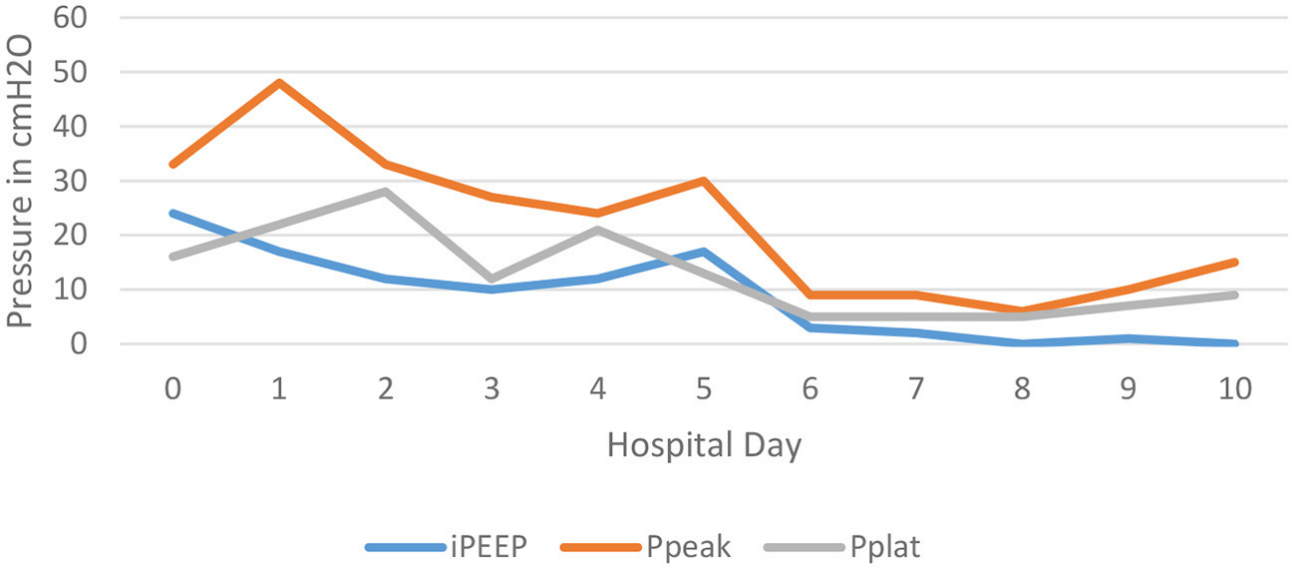
This depicts the trend of the patient's intrinsic PEEP, peak airway pressures, and plateau pressures over the first 10 days of the hospitalization. Notice the significant decrease in all three pressures after day 5; the day that isoflurane was administered.

On the morning after initiation, there was clinical improvement in air movement and markedly reduced airway resistance. The intrinsic PEEP was noted to be 3–4 cm H_2_O, see Figure 1. Two days after isoflurane therapy, the patient's tidal volume (500 mL) and respiratory rate (20 bpm) were able to be increased without development of elevated airway pressures or intrinsic PEEP, allowing for weaning of ECMO support. He was decannulated from ECMO on hospital day 10, and extubated hospital day 17, see Table 1.

**Table 1:** Laboratory values at the time of admission, immediately prior to and after ECMO cannulation, mid-ECMO run, day of decannulation from ECMO, day of extubation, and day of discharge.

	**Admission**	**Pre-cannulation**	**Post cannulation**	**Mid-ECMO Run**	**Decannulation**	**Extubation**	**Discharge**
WBC count	19.8	23.07	18.76	15.7	17.5	9.5	6.93
Hemoglobin	13.4	13.8	10.6	9.4	8.4	8.0	11.0
BUN	29	31	46	36	28	20	6
Creatinine	1.19	1.42	1.24	0.64	0.46	0.41	0.45
pH	7.17	7.17	7.35	7.41	7.40		
pCO_2_	>95	>95	77	69	49		
pO_2_	77	81	272	81	88		

WBC count measured in 10^*^3/μL, Hemoglobin is measured in g/dL, BUN and Creatinine are measured in mg/dL, pCO_2_ and pO_2_ are measured in mmHg. WBC, White Blood Cell; BUN, Blood Urea Nitrogen; pCO_2_, Partial pressure of arterial carbon dioxide; pO_2_, Partial pressure of arterial oxygen.

Although during the admission he only met 2 of the primary criteria for allergic bronchopulmonary aspergillosis, the patient was started on a prolonged course of isovuconazole as he grew aspergillus in his sputum, had tree-in-bud opacities in left lower lobe on CT of the chest, and was having intermittent fevers. His IgE level was normal at 109 kU/L, but it was noted that patient had been on high-dose systemic steroids since admission. He was discharged to an acute rehabilitation facility on Isovuconazole therapy, with plans to continue this for 12 weeks, and follow up with the infectious disease specialists in the outpatient setting with interval imaging as well.

## Discussion

This case illustrates the successful use of isoflurance delivered systemically through the ECMO circuit in a patient with status asthmaticus. Previous reports describe administration of isoflurane solely to the bronchiolar system *via* inhalation. This alternative route of delivery was chosen due to the inability to utilize inhaled anesthetics in the intensive care unit (safety concerns) and the inability to transport the patient to the operating room for delivery the anesthetic (resource limitation). Systemic delivery of volatile anesthetics could still lead to gas exposure to the room from the ventilator exhaust, it likely would be minimal and significantly less than previously describes delivery routes.

According to the Extracorporeal Life Support Organization (ELSO) Registry, a total of 272 patients were placed on ECMO for management of life-threatening asthma from March 1992 to March 2016. In this group, the weaning success rate was 86.7%, and the rate to survival to hospital discharge was 83.5% (25). A small case series (24 patients) sites mortality of roughly 16% for patients requiring VV-ECMO for SA (13). Commonly encountered complications include major bleeding (due to coagulopathy or the utilization of systemic anticoagulation to prevent thromboses), thrombosis, infection, renal failure, pulmonary and CNS hemorrhage (13).

Volatile anesthetics cause relaxation through direct action on bronchiole smooth muscle and through systemic uptake (19). They have also shown immune modulatory and anti-inflammatory actions (26). The use of volatile anesthetics in management of refractory status asthmaticus has been described since the 1980's. In most cases, these agents are delivered *via* inhalation, solely to the bronchiolar system. In fact, to our knowledge, we report the first case of isoflurane delivered systemically *via* the ECMO circuit as rescue therapy for severe refractory status asthmaticus. There is a higher risk for hypotension due to dose-dependent reduction in systemic vascular resistance and cardiac arrhythmias when used intravenously. This was not witnessed in the case of our patient.

The coordination of care in this case was complex, and the time spent by multiple providers, both in consultation with each other and individually, would likely limit the possibility of isoflurane delivery becoming a common practice. Anesthetic delivery systems that administer volatile anesthetics directly into endotracheal tubes have been developed and are being researched for the delivery of anesthetics to invasively ventilated patients (22). In a retrospective study of 74 patients, Grasselli et al. found that inhaled volatile anesthetic (isoflurane) delivered *via* the inhalation route using one of these anesthetic delivery systems can be a safe alternative to continuous IV sedation in patients on VV ECMO for ARDS. While systemic delivery is novel, there are limitations that should be mentioned. The amount of systemic absorption is unknown and dependent on the size of the gas used as well as the oxygenator components (the type of plastic used and how porous it is). The long-term use of these gases in the treatment of status asthmaticus is also unknown. Larger scale studies and more case reports are necessary to further evaluate the use of inhaled volatile anesthetics in patients on VV ECMO, and for management of severe refractory status asthmaticus in adults.

## Data availability statement

The original contributions presented in the study are included in the article/Supplementary material, further inquiries can be directed to the corresponding author.

## Ethics statement

Ethical review and approval was not required for the study on human participants in accordance with the local legislation and institutional requirements. Written informed consent from the (patients/participants OR patients/participants legal guardian/next of kin) was not required to participate in this study in accordance with the national legislation and the institutional requirements. Written informed consent was obtained from the patient for the publication of any potentially identifiable images or data included in this article.

## Author contributions

BG wrote the manuscript, developed the tables and figures, and researched the relevant information. AG contributed to the manuscript, made several revisions, and revised figures and tables. JB, ED, and NP revised the manuscript and contributed to writing. All authors contributed to the article and approved the submitted version.

## Conflict of interest

The authors declare that the research was conducted in the absence of any commercial or financial relationships that could be construed as a potential conflict of interest.

## Publisher's note

All claims expressed in this article are solely those of the authors and do not necessarily represent those of their affiliated organizations, or those of the publisher, the editors and the reviewers. Any product that may be evaluated in this article, or claim that may be made by its manufacturer, is not guaranteed or endorsed by the publisher.
